# α1胸腺肽减轻放射性肺损伤

**DOI:** 10.3779/j.issn.1009-3419.2011.03.02

**Published:** 2011-03-20

**Authors:** 荣 余, 宇 孙, 庆 蔡, 永恒 李, 广迎 朱

**Affiliations:** 1 100142 北京，北京大学临床肿瘤学院，北京肿瘤医院暨北京市肿瘤防治研究所放射治疗科，恶性肿瘤发病机制及转化研究教育部重点实验室 Key Laboratory of Carcinogenesis and Translational Research (Ministry of Education), Department of Radiation Oncology, Peking University School of Oncology, Beijing Cancer Hospital and Institute, Beijing 100142, China; 2 100142 北京，北京大学临床肿瘤学院，北京肿瘤医院暨北京市肿瘤防治研究所病理科 Department of Pathology, Peking University School of Oncology, Beijing Cancer Hospital and Institute, Beijing 100142, China; 3 100142 北京，中国人民解放军空军总医院检验科 Department of Laboratory Examination, the General Hospital of Air Force PLA, Beijing 100142, China

**Keywords:** 放射性肺损伤, α1胸腺肽, 小鼠, Radiotherapy lung injury, Thymosin alpha-1, Mouse

## Abstract

**背景与目的:**

放射性肺损伤是限制肺癌放疗疗效提高的主要因素之一，胸腺肽与放疗同时使用是否会加重放射性肺损伤尚不明确。本研究旨在用小鼠的放射性肺损伤模式评价α1胸腺肽对放射性肺损伤的影响。

**方法:**

33只雌性C57BL/6J鼠，体重19 g左右，分为正常空白对照（C）组、单纯照射（RT）组、α1胸腺肽加照射（T+RT）组。用小鼠死亡比例、体重、胸水、肺系数、肺泡灌洗液蛋白含量和细胞计数、肺泡壁肿胀和细胞浸润及肺纤维化积分作为观察指标来评价三组间的差异。

**结果:**

T+RT组与RT组小鼠死亡比例分别为3/14、2/10，死亡时间均为23周-24周。第24周时解剖发现RT组有一侧大量胸腔积液，一侧中等量积液，T+RT组未见明显胸腔积液。T+RT组肺系数、肺泡灌洗液蛋白含量和细胞总数、中性粒细胞计数均在第8周低于RT组，但巨噬细胞数第8周时T+RT组高于RT组。第24周T+RT组肺系数、肺泡灌洗液蛋白含量和细胞总数、中性粒细胞数、肺泡壁肿胀和细胞浸润及肺纤维化积分均明显低于RT组。C组肺泡灌洗液蛋白含量和细胞总数、肺泡壁肿胀与炎性细胞浸润均较低，未发生明显肺纤维化。

**结论:**

α1胸腺肽可能具有一定的防治放射性肺损伤的作用。

## Abstract (en)

**Background and objective:**

Radiation-induced lung injure is one of the major factors of limitation in radiotherapy for lung cancer. Whether the use of thymosin and radiotherapy simultaneously would increase the radiationinduced lung injure is unclear. The aim of this study is to evaluate the effects of thymosin alpha-1 on radiation induced pneumonitis in mice.

**Methods:**

Three groups of mice, control (C), radiation alone (RT), thymosin alpha-1 plus radiation (T+RT), were entered into the study. The weight and mortality of mice, pleural effusion, quantity of protein and cell count in the bronchoalvealar lavage (BAL) and pulmonary fibrosis score were evaluated as the outcome measures.

**Results:**

The mortality ratio of the T+RT and RT groups were 3/14, 2/10, respectively. The time of death were all in the 23-24 weeks after radiotherapy. There was no pleural effusion in the T+RT group other than 2/2 occured in RT group. The quantity of protein, cell number and neutrophil number in the BAL and lung coefficient in mice of T+RT group were remarkably lower than that of RT group, but the BALF macrophages number was remarkably higher than that in RT group in the 8 weeks. The quantity of protein, cell number, neutrophil number and macrophages number in the BAL, lung coefficient, the scores of lung fibrosis in mice of T+RT group were significantly lower than that of RT group in the 24 weeks. All test data were lowest in mice of C group. And there was no obvious pulmonary fibrosis in the mice of C group.

**Conclusion:**

Thymosin alpha-1 could relieve radiation-induced acute and late pulmonary injuries.

随着工业化程度的提高，大气污染日益严重，肺癌在许多国家和地区的发病率和死亡率均呈上升趋势。在恶性肿瘤死因中死于肺癌者，男性居首位，女性居第2位或第3位，是严重威胁人类健康和生命的常见恶性肿瘤^[1]^。随着精确放疗技术、放化综合治疗技术的进步，肺癌放疗疗效越来越高，如日本早期肺癌的放疗后5年生存率高达70%-80%^[2]^，完全可以和手术相媲美。美国和欧洲大规模临床随机研究^[3-5]^也证明：原发灶较大及肺门、纵隔淋巴结转移的局部晚期非小细胞肺癌（non-small cell lung cancer, NSCLC）放化综合治疗的疗效也与手术相似，放疗在肺癌治疗中的作用十分重要。放射性肺炎是肺癌放疗的常见并发症，也是限制肺癌放疗疗效进一步提高的主要因素之一。因此，如何避免和减少放射性肺炎的发生是肺癌治疗策略的重要组成部分。α1胸腺肽（商品名日达仙）为美国赛生药品股份公司研制的一种人工合成的胸腺素thymosin alpha-1，是一种较为有效的免疫功能生物反应调节剂，且副反应轻微。α1胸腺肽有明显的免疫刺激作用和直接的抗病毒、抗肿瘤作用，被广泛应用于治疗肿瘤和免疫缺陷性疾病（慢性病毒性肝炎、获得性免疫缺陷综合症等）。在放疗和化疗后应用α1胸腺肽可明显提高机体免疫状态，降低感染率，改善生活质量。但胸腺肽与放疗同时使用是否会加重放射性肺损伤，目前相关报道很少。本研究通过小鼠放射性肺损伤模型观察照射与α1胸腺肽结合的肺损伤情况，以探讨α1胸腺肽能否减轻或加重放射性肺炎。

## 1 材料与方法

### 1.1 实验动物

将7周-8周雌性C57BL/6J小鼠（19 g左右）饲养一周，使其充分适应环境。分入正常空白对照（C）组、单纯照射（RT）组、α1胸腺肽加照射（T+RT）组，共3组。C组每个观察点3只，RT组前2个观察点各3只，最后一个观察点4只；T+RT组前2个观察点各4只，最后一个观察点6只；共33只。

### 1.2 实验方法

非麻醉状态下固定小鼠，用6MV-X线、2 cm的放射野照射小鼠胸腔，屏蔽其它部分；照射剂量为15 Gy单次完成。3只-5只同时照射，加4 cm的填充物调整小鼠之间照射剂量的均匀性，照射前用剂量计实测剂量。α1胸腺肽加照射（T+RT）组用α1胸腺肽200 μg/kg腹腔注射，1次/天，连续10 d，于第5天用6MV-X线照射小鼠全肺15 Gy单次完成。α1胸腺肽（美国赛生，粉针剂，3.2 mg/支）用77 mL 0.9%生理盐水稀释，浓度为40 μg/mL（200 μg/kg·只）。小鼠购于中国医学科学院动物研究所，饲养在北京大学临床肿瘤学院清洁动物室，3只/笼-5只/笼。

### 1.3 实验观察

每月记录小鼠体重至少1次。实验后第3、8、24周为观察点，在每个观察点终止观察各组3只-4只小鼠。小鼠处死后先开胸观察是否有胸水，而后解剖游离全肺，用1 mL生理盐水连续3次对全肺进行肺泡灌洗，收集肺泡灌洗液并记录其量，用于细胞数和蛋白含量检测；全肺福尔马林固定24 h，石蜡包埋切片后给予HE染色，在光学显微镜下评价肺泡壁肿胀和炎性细胞浸润及肺纤维化情况。HE染色片在切片观察者不知入组情况下进行，每标本评分2张以上切片，随机4个以上视野，计算平均值和标准差。肺泡壁肿胀和炎性细胞浸润评分0分-4分：0分为正常。1分为肺泡壁稍增宽；有少许炎性细胞浸润，高倍视野下 < 10个炎性细胞。2分为肺泡壁明显增宽；有明显炎性细胞浸润，高倍视野下10个-20个炎性细胞。3分为肺泡壁明显增宽；炎性细胞浸润布满肺泡壁。4分为除炎性细胞浸润布满肺泡壁外，明显的肺泡内浸润。在切片观察者不知入组情况下进行，每标本评分2张以上切片，随机4个以上视野，计算平均值和标准差。肺纤维化积分为0分-8分（正常-严重）：0分为正常；1分为肺泡或细支气管壁少许纤维化；2分-3分为中度纤维化而无结构改变；4分-5分有明确的肺泡结构改变和纤维小结；6分-7分为严重肺泡结构改变和大片纤维化；8分为完全纤维化^[4]^。

### 1.4 实验检测

开胸取肺，全肺均在主支气管上环甲膜处离断，滤纸吸去肺表面血水，电子天平称重，肺湿重除以鼠体重乘以100得肺系数^[7]^。

用PRO-MEASURE蛋白定量试剂检测肺泡灌洗液蛋白含量，步骤与方法如下：先取100 μL待测样本置入不同清洁试管内，加入1 mL稀释的PRO-MEASURE溶液，室温温育2 min，595 nm处测吸光度值，通过公式Y（蛋白浓度）=40.277×A_595_+2.930计算肺泡灌洗液蛋白含量（mg/mL）。肺泡灌洗液细胞计数为镜下细胞计数板上进行，每样本检测2次，计算每毫升肺泡灌洗液的细胞数及分类。

### 1.5 统计方法

采用Primer统计软件进行数据分析，采用卡方检验分析各组小鼠死亡数以及出现胸水情况的差异，采用*t*检验分析各组间小鼠体重变化、肺系数、肺纤维化积分和肺泡灌洗液中细胞数及蛋白含量差异，*P* < 0.05为差异有统计学意义。

## 2 结果

### 2.1 死亡情况

T+RT组和RT组小鼠因死亡比例分别为3/14、2/10，T+RT、RT组间差异无统计学意义（*χ*^2^=2.219, *P*=0.330）。

### 2.2 小鼠一般情况的变化

C组的体重稳定增加。T+RT组与RT组增加幅度较空白对照组低，并于照射后第20周开始小鼠出现不同程度呼吸加快，活动减少，食欲下降，体重下降。T+RT组于20周开始下降，24周时体重有回升。RT组24周明显下降（*P*=0.04），但T+RT组与RT组相比差异无统计学意义。

### 2.3 肺大体改变

在24周终止观察时，RT组2只均伴有一侧大量胸腔积液及一侧中等量胸腔积液，而T+RT组和对照组均无明显胸腔积液（*χ*^2^=3.76, *P*=0.05）。RT组肺系数在第8周、第24周均明显高于T+RT组，详见图 1。

**1:**
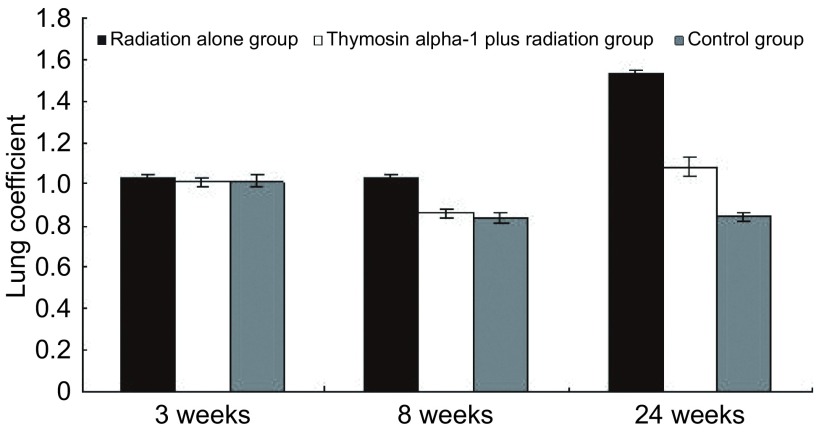
各组小鼠不同时间肺系数变化情况 Mice lung coefficient changes in each group at different time

### 2.4 肺泡灌洗液蛋白含量

第3周开始T+RT组与RT组肺泡灌洗液蛋白含量明显高于对照组，第3周T+RT组为（2.04±0.72）mg/mL，RT组为（2.33±0.46）mg/mL，组间无差异（*t*=0.66, *P*=0.54）；第8周T+RT组为（2.13± 0.24）mg/mL，低于RT组（3.53±1.27）mg/mL（*t*=1.94, *P*=0.11），第24周T+RT组为（1.69±0.72）mg/mL，明显低于RT组（3.56±0.19）mg/mL（*t*=3.43, *P*=0.04），详见图 2。

**2:**
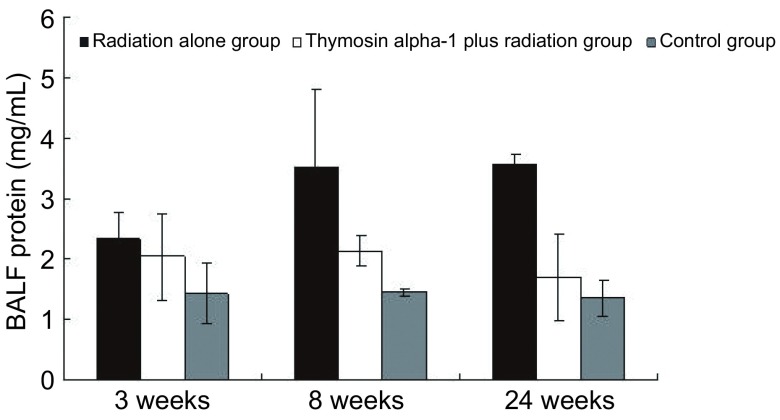
各组小鼠不同时间肺泡灌洗液蛋白量变化情况 Mice bronchoalveolar lavage fluid protein (BALF) changes in each group at different time

### 2.5 肺泡灌洗液细胞数

第3周T+RT组与RT组的肺泡灌洗液细胞总数、巨噬细胞数、中性粒细胞数分别是50.00±8.64与51.33±37.81（*t*=0.06, *P*=0.96）、7.50±4.43与16.67±16.77（*t*=0.92, *P*=0.45）、24.50±7.19与25.00±16.70（*t*=0.055, *P*=0.96）。RT组肺泡灌洗液细胞总数、中性粒细胞数均在第8周高于T+RT组，但巨噬细胞数第8周时T+RT组高于RT组。第8周T+RT组与RT组的肺泡灌洗液细胞总数、巨噬细胞数、中性粒细胞数分别是400.50 ±109.12与609.00±285.93（*t*=1.20, *P*=0.33）、121.50±285.93与87.00±88.79（*t*=-0.73, *P*=0.50）、186.75±62.52与399.00 ±189.71（*t*=1.86, *P*=0.19）。第24周T+RT组与RT组的肺泡灌洗液细胞总数、巨噬细胞数、中性粒细胞数分别是19.33±9.87与202.50±6.36（*t*=22.66, *P* < 0.01）、6.67±1.15与72.00±12.73（*t*=9.66, *P* < 0.01）、9.33±6.43与49.50±6.36（*t*=6.87, *P* < 0.01）。第24周RT组肺泡灌洗液细胞总数、中性粒细胞数均明显高于T+RT组。巨噬细胞数变化情况详见图 3。

**3:**
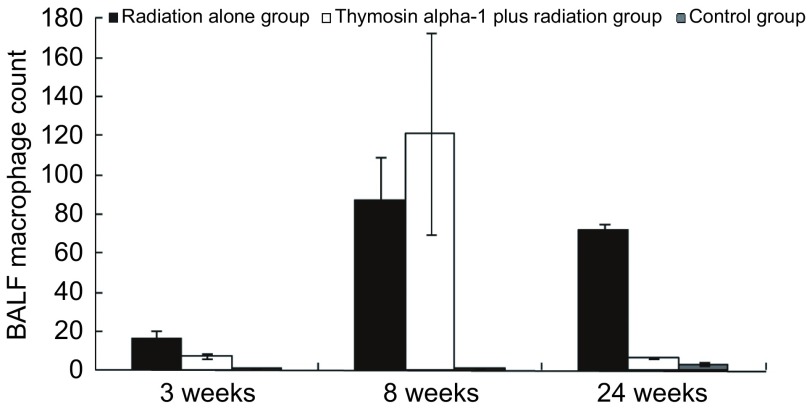
各组小鼠不同时间巨噬细胞数变化情况 Mice bronchoalveolar lavage fluid (BALF) macrophages number changes in each group at different time

### 2.6 各观察点的鼠肺泡壁肿胀与炎性细胞浸润及纤维化评分

光镜观察C组大鼠支气管、肺泡及肺泡间隔组织结构正常，无充血、水肿及急慢性炎症等改变。RT组小鼠第3周肺毛细血管充血，肺泡间隔增宽且水肿，可见中性粒细胞和单核巨噬细胞浸润；第8周肺泡间隔进一步增宽，纤维细胞和胶原纤维增生；有大量中性粒细胞和单核巨噬细胞浸润，小鼠肺中等支气管及血管周围均有炎性细胞聚集；第24周，部分肺泡腔消失，萎缩，肺泡壁断裂，肺泡腔内可见脱落的肺泡上皮及蛋白渗出液。肺泡隔明显增宽，炎症细胞浸润较第8周时减轻，纤维细胞增生及胶原纤维沉积在肺泡间隔内肺泡炎明显。T+RT组小鼠第3周肺毛细血管充血，肺泡间隔增宽且水肿，也可见中性粒细胞和单核巨噬细胞浸润；第8周肺泡间隔进一步增宽，纤维细胞和胶原纤维增生，有大量中性粒细胞和单核巨噬细胞浸润，小鼠肺中等支气管及血管周围均有炎性细胞聚集；第24周，肺呈正常结构，有区域性点灶性纤维化结节，肺泡腔内可见脱落的肺泡上皮及蛋白渗出液，肺泡隔增宽，炎症细胞浸润较第8周时减轻，纤维细胞增生及胶原纤维沉积在肺泡间隔内，肺泡炎明显，第24周时T+RT组的肺泡壁肿胀与炎性细胞浸润评分和肺纤维化积分明显低于RT组，*P*值分别为0.04和0.03，有统计学差异。详见表 1、表 2，图 4。

**1:** 小鼠各观察点的肺泡壁肿胀与炎性细胞浸润评分 Mice alveolar wall swelling and inflammatory cell infiltration score in each group at different time

Time	RT group (*n* =10)	T+RT group (*n* =14)	Control group (*n* =9)	t^*^	P^*^
3 weeks	0	0.50±0.58	0	-1.46	0.20
8 weeks	0.33±0.58	1.00±0.82	0	-1.27	0.26
24 weeks	2.00±1.41	0.67±0.58	0	1.55	0.04
RT: radiation alone; T+RT: thymosin alpha-1 plus radiation. ^*^Statistical analysis on RT group and T+RT group.					

**2:** 小鼠各观察点的肺纤维化积分 Mice points of pulmonary fibrosis in each group at different time

Time	RT group (*n* =10)	T+RT group (*n* =14)	Control group (*n* =9)	t^*^	P^*^
3 weeks	0	0.50±0.58	0	-1.46	0.20
8 weeks	0	0.50±0.58	0	-1.46	0.20
24 weeks	4.00±1.41	0.67±0.58	0	3.87	0.03
^*^Statistical analysis on RT group and T+RT group.					

**4:**
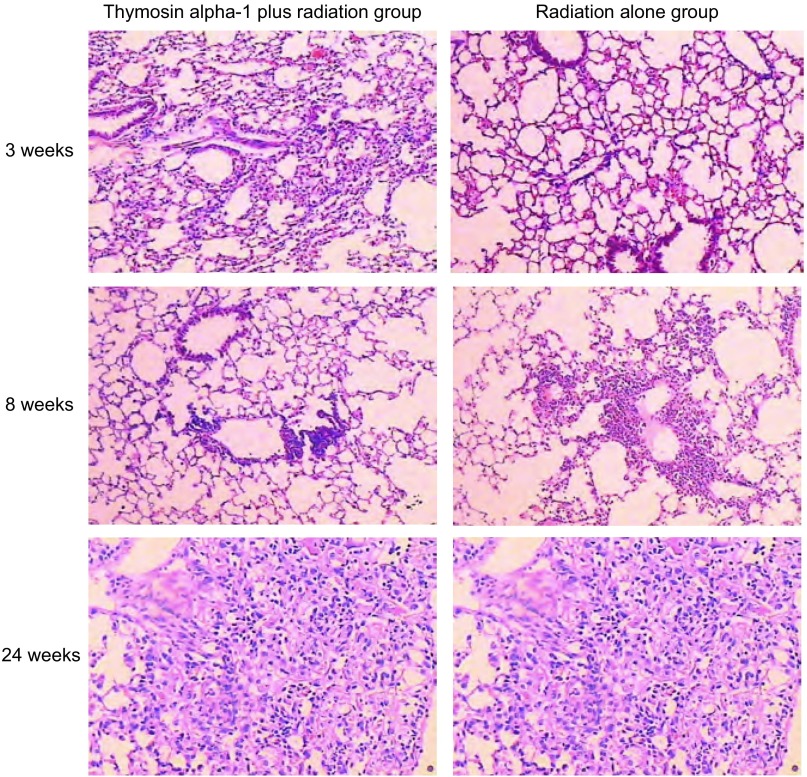
各组小鼠不同时间肺泡壁肿胀与炎性细胞浸润、肺纤维化情况（HE, ×20） Mice alveolar wall swelling, inflammatory cell infiltration and pulmonary fibrosis in each group at different time (HE, ×20)

## 3 讨论

本研究采用动物模型经小鼠胸水、肺系数、病理组织学肺泡壁肿胀与炎性细胞浸润评分和肺纤维化积分、肺泡灌洗液的细胞计数、蛋白定量分析，初步证明α1胸腺肽可明显减轻放射性肺损伤。从小鼠一般情况、肺的大体改变看，本研究显示在第20-24周时RT组小鼠的体重明显下降，说明此期间小鼠生活质量明显下降，而T+RT组在第16-20周时体重已开始下降，但在第20-24周时体重回升，说明虽然放疗使小鼠生活质量下降，但α1胸腺肽未加重其影响，反而对小鼠的生活质量有一定改善。但各时点体重变化没有统计学差异，这可能与实验的样本量小有关。24周时，RT组存活的2只小鼠均产生了大量胸腔积液，而T+RT组存活的3只小鼠均未见明显胸腔积液，胸水产生的只数比具有统计学差异。此外，第8周、第24周T+RT组的肺系数均明显低于RT组。在肺纤维化的形成过程中由于细胞水肿、炎性渗出、毛细血管充血等因素造成的肺重量增加，是肺系数升高的直接原因。从上述表现可见α1胸腺肽对放射性肺损伤的保护趋势。

长期以来，胸部肿瘤在放疗或放化综合治疗时，急性或亚急性放射性肺炎和晚期肺纤维化是最常见的并发症，严重阻止了通过放疗根治癌细胞的机会，增加剂量和肺的治疗体积以及合并化疗明显增加放射性肺损伤的几率^[8]^。导致肺损伤的细胞与分子生物学机制目前比较公认的是Tsoutsou等^[9]^的研究观点，即在辐射导致肺损伤后，早期开始不断出现一些类似激素样作用的细胞因子在肺组织间质细胞（炎细胞、组织特异功能细胞和纤维母细胞等）损伤中发挥重要作用，这些细胞因子长期的通过自分泌、旁分泌和内分泌的连锁反应导致早期的炎症和晚期肺纤维化，最后表现为明显的病理改变和临床症状。

α1胸腺肽的是人工合成的胸腺肽，具有诱导T细胞分泌，促进T淋巴细胞亚群发育、成熟并活化的功能，能调节T淋巴细胞亚群的比例，并能增强巨噬细胞的吞噬功能，提高自然杀伤细胞活力，提高IL-1的产生与受体表达水平^[10]^。在临床研究方面，周建平^[11]^报道的随机对照研究中，30例胸腔内灌注化疗药联合胸腺肽α1疗效明显优于单纯胸腔内灌注化疗药，并能改善临床症状，提高免疫功能，是肺癌并胸腔积液有效治疗方法之一。王慧敏等^[12]^采用随机、对照、前瞻研究胸腺肽α1提高Ⅲ期-Ⅳ期NSCLC化疗疗效的作用，共40例，发现与单纯化疗组相比，胸腺肽α1可提高生活质量和生存率，且无明显的毒副作用，安全、有效。Garaci等^[13]^对随机分组的56例晚期NSCLC患者用顺铂（DDP）、鬼臼乙叉甙（VP-16）、胸腺肽Tα1（1 mg/d, d8-11, d15-18）和小剂量IFN（300万IU, im, d11, d18），21 d为一疗程。在56例完成治疗的患者中有24例有效（2例CR，22例PR），总有效率为42.9%，中位生存时间为12.6个月。在联合治疗组未发现明显的毒性反应。免疫功能监测结果表明：单纯DDP+VP-16进行化疗后，NK细胞活性和淋巴细胞数量均明显下降，而联用胸腺肽和IFN后，免疫抑制受到明显改善。另一临床试验^[14]^结果表明：在随机采用异环磷酰胺联合胸腺肽和小剂量IFN治疗的22例晚期NSCLC中，有效率为33%，单用异环磷酰胺化疗组有效率仅为10%，而且联合治疗组的毒副作用明显下降，患者的耐受性明显增加，并认为其作用机制可能在于Tα1刺激了直接针对肿瘤细胞抗原的特殊淋巴单核细胞系。Garaci等^[15]^2003年再次报道Tα1可以明显增加细胞因子的活性，并能减轻细胞毒性药物的血液毒性。另外An等^[16]^报道了Tα1对22例进展期肺癌或乳腺癌患者化疗所致神经毒性有保护作用。但胸腺肽与放疗同时使用是否会加重放射性肺损伤，目前尚无相关报道。本研究发现实验处理后小鼠胸水、肺系数、肺泡灌洗液的细胞数与蛋白含量和肺纤维化积分均显示T+RT组比RT组的肺组织损伤明显减轻。

放射性肺损伤与修复是一连续的损伤与修复交替并存的复杂过程。肺组织受到一定剂量照射后，肺泡II型细胞发生改变，3周-4周恢复正常，导致肺泡内表面活性物质增多^[17]^。早期肺泡灌洗液内蛋白成分轻微升高，可不伴细胞成分，而后逐渐恢复正常，这些早期改变是局部组织早期损伤反应，可能是电离辐射直接造成的。随后机体免疫反应或炎性反应发生，肺泡灌洗液中蛋白含量、中性粒细胞和淋巴细胞明显升高。Inoue等^[18]^研究发现无特定病原体Wistar雄鼠双肺接受18 Gy照射后28天通过马森三染色可见极少量肺泡壁纤维化，照射后56天通过马森三染色可见相对弥漫的肺泡壁纤维化，照射后巨噬细胞、中性粒细胞和淋巴细胞开始升高，分别在第28天、第56天达高峰，蛋白含量升高。许多文献^[19-24]^报道照射后早期1周-2周内蛋白成分无明显增加，照射后1个月左右蛋白和细胞成分才开始升高。本研究结果与之类似，肺泡灌洗液内蛋白和细胞成分从第3周开始升高，第3周时T+RT组与RT组并无明显差异。在第8周RT组肺泡灌洗液细胞总数、中性粒细胞数均高于T+RT组，可见α1胸腺肽并未加重炎症反应，反而有减轻反应的趋势。在第24周RT组肺泡灌洗液细胞数、蛋白含量均明显高于T+RT组，初步显示出α1胸腺肽对放射性肺损伤的保护作用。

从病理变化来看，急性放射性肺炎其特征为肺泡萎陷、膨胀不全及血管内物质渗入肺泡腔，肺泡Ⅱ型细胞细胞浆内板层小体减少，细胞变形、肿胀脱落入肺泡腔内，血管内皮细胞超微结构改变，细胞呈多型性，细胞内空泡形成，内皮细胞脱落，血管渗透性增加，这提示Ⅱ型肺泡上皮细胞和血管内皮细胞有可能是放射性肺损伤的靶细胞。此时可以出现咳嗽、呼吸困难、发热等症状，但也可以没有临床表现。经过一段时间后，临床症状可能有所减轻，但组织学改变将继续发展，胶原沉积，毛细血管闭塞，肺泡间隔增厚，逐渐进入纤维化期^[25]^。但至今为止，没有发现任何一个因子可以对致病过程起独立的决定性作用，这显示肺的放射性损伤过程实质上是一个复杂的多方面的过程，是由涉及到的多种因子相互作用所引起的一个长期的过程。这些持续存在的细胞因子相互作用的逐渐增强，导致了致纤维化细胞因子的表达，最后产生了晚期损伤的特征性表现纤维化^[26]^。本研究中观察到小鼠肺部的变化与上述描述相符，RT组小鼠第3周肺毛细血管充血，肺泡间隔增宽且水肿，可见中性粒细胞和单核巨噬细胞浸润，第8周肺泡间隔进一步增宽，纤维细胞和胶原纤维增生；有大量中性粒细胞和单核巨噬细胞浸润，小鼠肺中等支气管及血管周围均有炎性细胞聚集，第24周部分肺泡腔消失、萎缩，肺泡壁断裂，肺泡腔内可见脱落的肺泡上皮及蛋白渗出液。肺泡隔明显增宽，炎症细胞浸润较第8周时减轻，纤维细胞增生及胶原纤维沉积在肺泡间隔内肺泡炎明显。T+RT组小鼠的变化与单纯放疗组相似，但第24周时，仍可见肺部呈正常结构，有区域性点灶性纤维化结节，肺泡腔内可见脱落的肺泡上皮及蛋白渗出液，肺损伤明显轻于RT组，进一步显示了α1胸腺肽对放射性肺损伤的防治作用。

肺泡巨噬细胞是肺脏的重要细胞，不但参与肺的防御及免疫，而且能分泌多种生物活性物质。慢性炎症中，巨噬细胞聚集并持续存在。其来源主要是从血液循环中不断募集单核细胞，离开血流游出血管后的单核细胞，转变为巨噬细胞。这是粘附分子和趋化因子持续稳定表达的结果。有文献^[27]^报道，局部一次大剂量照射后2周-3周，肺巨噬细胞数明显下降，4周-8周逐渐恢复到正常水平。本实验观察到RT组照射后24周时，大量肺泡腔可见巨噬细胞，大量出现较T+RT组晚，也许与射线在早期引起巨噬细胞损伤，而α1胸腺肽能诱导T细胞分泌，促进T淋巴细胞亚群发育、成熟并活化，同时调节T淋巴细胞亚群的比例，并增强巨噬细胞的吞噬功能有关。巨噬细胞激活后释放的成纤维细胞生长因子能够促进成纤维细胞的增生，并合成和分泌大量的胶原纤维使肺组织纤维化^[28]^。这可能是引起放射性肺纤维化的重要原因之一。本研究发现，α1胸腺肽并没有加重其炎症反应，反而减轻了放射性肺损伤，但其具体机制尚不明确。

总之，本研究采用动物模型经小鼠胸水、肺系数、病理组织学肺泡壁肿胀与炎性细胞浸润评分和肺纤维化积分、肺泡灌洗液的细胞计数、蛋白定量分析，初步证明α1胸腺肽与照射同期使用可明显减轻放射性肺损伤的发生，确切结论还有待进一步研究。
